# Shen-Zhi-Ling Oral Liquid Improves Behavioral and Psychological Symptoms of Dementia in Alzheimer's Disease

**DOI:** 10.1155/2014/913687

**Published:** 2014-05-18

**Authors:** Weidong Pan, Qiudong Wang, Shin Kwak, Yu Song, Baofeng Qin, Mingzhe Wang, Yoshiharu Yamamoto

**Affiliations:** ^1^Department of Neurology, Shuguang Hospital Affiliated to Shanghai University of TCM, 528 Zhang-Heng Road, Pu-Dong New Area, Shanghai 201203, China; ^2^Department of Neurology, Pudong New Area Hospital of Traditional Chinese Medicine, 460 Xiuchuan Road, Pu-Dong New Area, Shanghai 201200, China; ^3^Center for Disease Biology and Integrative Medicine, Graduate School of Medicine, The University of Tokyo, 7-3-1 Hongo, Bunkyo-ku, Tokyo 113-8655, Japan; ^4^Educational Physiology Laboratory, Graduate School of Education, The University of Tokyo, 7-3-1 Hongo, Bunkyo-ku, Tokyo 113-0033, Japan

## Abstract

We evaluated the effects of the traditional Chinese medicine (TCM) Shen-Zhi-Ling oral liquid (SZL) on the behavioral and psychological symptoms of dementia (BPSD) in patients with Alzheimer's disease (AD). Among 98 patients with AD and BPSD enrolled (mean age, 57.2 ± 8.9 years old), 91 (*M* = 55, *F* = 36; mean age, 57.2 ± 9.7 years old) completed the study. Patients took either SZL (*n* = 45) or placebo granules (*n* = 46) in a double-blind manner for 20 weeks while maintaining other anticognitive medications unchanged. Changes in BPSD between week 0, week 10, week 20, and week 25 were assessed using the behavioral pathology in Alzheimer's disease (BEHAVE-AD) rating scale and the neuropsychiatric inventory (NPI), detrended fluctuation analysis (DFA) represented by diurnal activity (DA), evening activity (EA), and nocturnal activity (NA) according to actigraphic recordings. SZL but not placebo oral liquid delayed the development of BPSD significantly according to the changes in some of the clinical scores and the EA and NA parameters of DFA at week 20 compared with week 0. No side effects were observed in laboratory tests. The results indicate that SZL might delay the development of BPSD in AD patients and thus is a potentially suitable drug for long-term use.

## 1. Introduction

Cognitive deficits and behavioral and psychological symptoms of dementia (BPSD) are typical features of patients with dementia such as Alzheimer's disease (AD), vascular dementia (VD), and other forms of senile dementia [1]. The symptoms include agitation, aberrant motor behavior, anxiety, elation, irritability, depression, apathy, disinhibition, delusions, hallucinations, and sleep or appetite changes. BPSD constitute a major component of the dementia syndrome irrespective of its subtype. The current limits of the effectiveness of pharmacotherapies highlight the value in delaying the progression of the disease and the functional decline [2].

Herbal remedies have a long history of use (particularly in East Asian countries) for alleviating various symptoms and have been increasingly used as alternative medicines worldwide, including the United States [3]. Traditional Chinese medicines (TCM) ameliorate various symptoms, particularly ageing-related symptoms [4, 5], and hence are likely to be beneficial for neurodegeneration diseases such as Parkinson's disease and motor neuron disease [6–8]. Good compliance for long-term use with few side effects may be another merit of TCM suitable for AD patients.

In this study, we evaluated the effects of the TCM Shen-Zhi-Ling oral liquid (SZL) on the symptoms of BPSD in AD patients. Pan et al. adopted a recently developed method analyzing scores of wrist activity measured with a motion logger [9] and showed that analysis of diurnal activity (DA), nocturnal activity (NA), and evening activity (EA) may reflect the fluctuational degrees of BPSD and can provide a useful assessment of BPSD accompanied by clinical scores for AD. The aim of the present study was to evaluate the ameliorating effects of SZL on impaired BPSD of AD patients using the quantitative and objective parameters recorded by a* Micro-Mini-Motionlogger* (Ambulatory Monitoring Inc.).

## 2. Methods

### 2.1. Subjects

Subjects with Mini-Mental State Examination (MMSE) scores between 10 and 24 and satisfying the fourth edition of the Diagnostic and Statistical Manual of Mental Disorders DSM-IV-TR Fourth Edition (DSM-IV) for dementia from January 2010 to October 2013 at the Department of Neurology of Shuguang Hospital Affiliated to the Shanghai University of TCM were recruited into the study. We examined 198 patients who had been diagnosed with cognitive disorders; however, only 98 patients who suffered from AD (mean age ± SD, 57.2 ± 8.9 years old, mean duration of illness, 5.9 ± 5.1 years) were found to be suitable for this research. Current diagnostic options in living patients include a combination of clinical history, the exclusion of other causes of cognitive impairment, and cognitive and mental state examination [10]. Structural imaging techniques with computed tomography (CT), magnetic resonance imaging (MRI), single-photon emission computed tomography (SPECT), positron emission tomography (PET), and/or with clinical signs and symptoms were used as an aid to diagnosis and to help differentiate AD from other types of dementia, such as vascular dementia, frontal temporal dementia, and Parkinson's disease with dementia. The patients were randomly assigned to the SZL (*n* = 49, 64.27 ± 11.8) or placebo group (*n* = 49, 63.91 ± 13.9) (Table 1) and given random numbers by a study coordinator, who also encoded the drugs with matching random numbers. Neither the patients nor the researchers monitoring the outcome knew which patient was receiving which treatment, until the study was over and the random code was broken. Antidementia drug administration was not changed throughout the experiment. The study was approved by The Ethics Committee of Shuguang Hospital Affiliated to Shanghai University of TCM and performed under the principles outlined in the Declaration of Helsinki. All subjects provided informed consent in accordance with institutional requirements prior to participation in the study.

### 2.2. Additional Treatment

Sheng-Zhi-Ling oral liquid (SZL), the TCM used in this study, is an oral liquid consisting of 10 kinds of traditional Chinese medicine:* Codonopsis pilosula*,* Cassia Twig, Paeonia lactiflora*,* honey-fried Licorice root, Poria Cocos, Rhizoma Zingiberis, Radix Polygalae, Acorus tatarinowii*,* Ossa Draconis,* and* Concha* Ostreae. SZL is commonly used in treating for “insufficiency of vitality (Qi) and innutrition of the mind (heart)” in China. Placebo oral liquid consisted of one-tenth of the volume of SZL together with an added bitterant and it hadno TCM activity. Patients were instructed to take one bottle (10 cc, including 500 mg crude drug) of SZL or placebo soluble liquid (10 cc, including 50 mg crude drug) three times a day at least 30 min before or after the ingestion of other drugs for 20 consecutive weeks. The shape and color of SZL and the placebo oral liquid were very similar and could not be distinguished from one another by appearance or aqueous solution taste. SZL and the placebo oral liquids were made by Shandong Wohua Pharmaceuticals Co., Ltd. The trial was carried out as a randomized, double-blind, parallel group study.

### 2.3. Assessments

Behavioral pathology in Alzheimer's disease (BEHAVE-AD) [11]: BEHAVE-AD addresses delusions, hallucinations, activity disturbances, aggressiveness, diurnal rhythm disturbances, affective disturbances, and anxieties and phobias. The BEHAVE-AD scores of all patients were evaluated 4 times on the day before the actigraph recordings in the series time windows during the 25 weeks of follow-up by the same neurologists, such as before taking additional TCM medicine and in week 10, week 20, and week 25 (5 weeks after stopping the additional treatment).

Neuropsychiatric inventory (NPI) [12]: NPI was used to assess 10 behavioral disturbances occurring in patients: delusions, hallucinations, dysphoria, anxiety, agitation/aggression, euphoria, disinhibition, irritability/ability, apathy, and aberrant motor activity. The NPI scores were assessed based on information from the patients or caregivers using the same time windows as when evaluating BEHAVE-AD.

Analysis of actigraphy: all patients wore a small watch-type activity monitor equipped with a computer (*Micro-Mini-Motionlogger*, Ambulatory Monitoring, Inc., Ardsley, New York) on the wrist of their nondominant hand for 7 consecutive days in the series time windows (10 weeks each and then 5 weeks) during the 25-week follow-up. Data acquired during the diurnal activity (DA, between 6 a.m. and 6 p.m.), evening activity (EA, between 6 p.m. and 9 p.m.), and nocturnal activity (NA, between 9 a.m. and 6 a.m.) periods were used in the analyses [9]. Discontinuous data were combined using an integrative method and then analyzed by detrended fluctuation analysis (DFA), which evaluates the correlations between time scales and magnitudes of fluctuation (standard deviations) within each time scale [13, 14]. We compared the fluctuation of these parameters in the series time windows during the 25 weeks of follow-up.

For the safety assessments, each patient underwent a physical examination by a physician and laboratory tests for blood counts and biochemistry and urinalysis at each visit.

### 2.4. Statistical Analysis

Repeated-measure ANOVA was conducted to test the differences among week 0, week 10, week 20, and week 25 for the scores of BEHAVE-AD, NPI, and the actigraph parameters in the SZL and placebo groups. When a significant difference was detected, a post hoc test (Bonferroni test) was conducted between the SZL and placebo groups. A significant difference was defined as *P* < 0.05. SPSS windows version 17.0 was used for statistical analyses. All data are expressed as the mean ± standard deviation.

## 3. Results

Seven patients dropped out of the study; two patients in the SZL group were unable to tolerate the bitter taste of SZL, while two in the SZL group and three in the placebo group dropped out due to a conflict with other TCM prescribed for concomitant diseases. Neither physical examination nor laboratory tests revealed any adverse changes after additional treatment in either group at the end of the study.

The post hoc test revealed no significant differences at baseline (week 0) and other each time point (week 10, week 20, and week 25) in age, duration of AD, MMSE, BEHAVE-AD, NPI scores, actigraph parameters, or in the dosages of huperzine A, aniracetam, memantine hydrochloride, donepezil hydrochloride, rivastigmine, and galantamine reminyl, between the SZL and placebo groups (Tables 1 and 2).

No significant changes were observed at week 10 by checking the clinical scores and DFA parameters for the two groups. When the effects of SZL at week 20 were evaluated by BEHAVE-AD scores, significant and persistent improvements were found in hallucinations, activity disturbances, aggressiveness, and anxieties and phobias compared with the placebo group. Except for paranoid and delusion ideation, all BEHAVE-AD scores at week 20 had improved in the placebo group compared with week 0, while there was little change in the SZL group. At week 20, half of the NPI mean scores, such as for delusions, hallucinations, agitation, aberrant motor behavior, and sleep disturbances in the SZL group, were much lower (less improvement) compared with the placebo group, while these scores in the placebo group were significantly higher at week 20 compared with those in the patients at week 0. Interestingly, the EA and NA scores for the DFA actigraph recordings showed significantly lower values for the SZL group compared with the placebo group at week 20, while these patients in the placebo group had significantly improved values compared with the DFA values at week 0, and the effects in the SZL group were maintained for 5 weeks at the endpoint of the research (Table 2). The appetites of patients in both the SZL and placebo groups at week 20 and week 25 showed almost no changes compared with week 0.

## 4. Discussion

We previously demonstrated that the changes of EA and NA in the DFA, which is in accordance with the improvement of the BEHAVE-AD and NPI scores, might be quantitative predictors for evaluating the severity of BPSD in dementia [9]. In this study, we demonstrate that SZL, a TCM, ameliorated the disability associated with BPSD in AD patients using the analysis of DFA of the actigraph records together with the more conventional BEHAVE-AD and NPI scores. SZL induced no significant adverse effects and was tolerable by more than 92% of the participants.

The treatment of BPSD is as important as the treatment of core symptoms such as memory disturbance and disorientation. Acetylcholinesterase (AChE) inhibitors and N-methyl-d-aspartate (NMDA) receptor noncompetitive antagonists are commonly used for the treatment of AD. They are effective at treating core symptoms and in BPSD treatment [15–17]; however, the effects are not completely satisfactory. They can cause adverse effects such as nausea, extrapyramidal symptoms, drowsiness, and other symptoms [18]. Many researchers have attempted to identify more effective medicines from traditional therapy or translational therapy for serious neurodegeneration disease [19–21]. The present preliminary data have replicated the previous finding that SZL could protect neurons by reducing the expression of APP mRNA in cerebral cortex and hippocampus and decreasing the expression of caspase-3 to reduce the apoptosis of neurons [22]. In TCM theory, insufficiency of body vitality (Qi) might cause abnormal body physical activity and a loss of the ability to control body movements. Innutrition of the mind (heart) may cause affective disorders and poor cognitive function and result in fatigue of the mind. These patients will present with hallucinations, activity disturbances, aggressiveness, diurnal rhythm disturbances, anxieties and phobias, agitation, aberrant motor behavior, depression, and even sleep disturbance [23]. Among the 10 components of SZL,* Codonopsis pilosula* and Cassia Twig might increase the vitality of the body and warm the body, thus providing more energy to control “Qi” properly [24, 25]. Radix Polygalaeand* Acorus tatarinowii *are both sedatives and heart invigorating and can also modify cognitive function [26, 27]. Ossa Draconis and Concha Ostreae are well-known sedatives, and researchers in many countries have demonstrated that they have a sleep-inducing function for treating sleep disorders [28]. The remaining herbs in the SZL concoction can increase blood circulation in the brain (*Paeonia lactiflora, *honey-fried Licorice root, and Poria Cocos) [29, 30]. Whether the components of SZL contain inhibitory effects on fibril formation has not been demonstrated. Although the groups consisted of only small numbers of patients that resulted in differences at baseline despite randomization, it was unlikely that this altered the outcome, given the magnitude of change from baseline with SZL treatment. SZL is tolerable for long-term administration and hence is likely a suitable choice as an additional drug for long-term control of the symptoms of BPSD for AD. DFA, which determines the deviations in 3 parameters (DA, EA, and NA) obtained by actigraph recordings, can be quantitatively used for assessing the severity of BPSD in patients suffering from AD. The small sample size is a limitation of our pilot study. In addition, normative data for both healthy elderly and BPSD patients need to be established. Actigraphy may be feasible and useful when predicting a prognosis or making therapeutic decisions related to patients with AD-BPSD.

## Figures and Tables

**Table 1:** Characteristics of the patients with Alzheimer's disease before and after additional treatment.

Characteristic	Shen-Zhi-Ling group			
	1st week (*n*)	10th week (*n*)	20th week (*n*)	25th week (*n*)
Age (years)	57.2 ± 9.7			
Sex (M/F)	28/17			
Duration of VD (years)	5.7 ± 4.9			
MMSE	13.4 ± 1.8	12.9 ± 3.1	12.0 ± 2.3	11.8 ± 1.6
Huperzine A (*μ*g/d)	323.30 ± 173.9 (32)	334.41 ± 169.3 (32)	319.29 ± 168.6 (31)	349.32 ± 188.4 (31)
Aniracetam (mg/d)	489.6 ± 179.3 (28)	491.7 ± 166.8 (28)	490.5 ± 183.4 (29)	497.6 ± 112.3 (29)
Memantine hydrochloride (mg/d)	6.67 ± 5.28 (28)	6.81 ± 4.47 (30)	6.83 ± 4.96 (29)	6.92 ± 5.67 (30)
Donepezil hydrochloride (mg/d)	8.21 ± 3.76 (16)	8.49 ± 4.02 (15)	8.73 ± 2.69 (15)	9.33 ± 6.94 (15)
Rivastigmine (mg/d)	3.38 ± 1.26 (23)	3.47 ± 1.72 (14)	3.55 ± 2.09 (13)	3.75 ± 1.66 (14)
Galantamine reminyl (mg/d)	25.89 ± 22.63 (26)	25.31 ± 23.61 (25)	25.87 ± 21.39 (26)	26.82 ± 22.91 (26)

Characteristic	Placebo group			
	1st week (*n*)	10th week (*n*)	20th week (*n*)	25th week (*n*)

Age (years)	56.9 ± 10.2			
Sex (M/F)	27/19			
Duration of VD (years)	5.9 ± 5.2			
MMSE	14.1 ± 1.5	13.3 ± 2.7	12.07 ± 3.5	11.2 ± 2.8
Huperzine A (*μ*g/d)	358.30 ± 191.4 (34)	334.41 ± 169.3 (34)	327.03 ± 179.8 (33)	358.30 ± 191.4 (32)
Aniracetam (mg/d)	506.6 ± 108.7 (26)	498.8 ± 171.2 (25)	505.5 ± 192.3 (27)	517.2 ± 119.3 (27)
Memantine hydrochloride (mg/d)	6.71 ± 4.47 (29)	6.67 ± 5.09 (30)	6.59 ± 5.09 (29)	6.98 ± 4.47 (30)
Donepezil hydrochloride (mg/d)	8.23 ± 6.74 (14)	8.37 ± 5.33 (15)	8.66 ± 2.71 (16)	8.92 ± 7.58 (15)
Rivastigmine (mg/d)	3.45 ± 1.65 (25)	3.51 ± 1.08 (24)	3.61 ± 1.87 (25)	3.67 ± 1.72 (25)
Galantamine reminyl (mg/d)	26.12 ± 22.84 (29)	26.08 ± 21.65 (29)	25.59 ± 20.41 (29)	27.51 ± 19.66 (29)

AD: Alzheimer's disease; MMSE: Mini-Mental State Examination.

**Table 2:** Results of clinical evaluation between before and after the additional treatment of Sheng-Zhi-Ling Oral liquid (SZL) and placebo.

Parameter	Week 0		Week 10		Week 20		Week 25	
	SZL	Placebo	SZL	Placebo	SZL	Placebo	SZL	Placebo
BEHAVE-AD								
Paranoid and delusion ideation	3.11 ± 0.38	3.06 ± 0.36	3.12 ± 0.29	3.26 ± 0.66	3.15 ± 0.71	3.31 ± 1.06	3.21 ± 1.04	3.49 ± 1.23
Hallucinations	3.72 ± 0.59	3.79 ± 0.61	3.75 ± 1.48	3.95 ± 0.61	3.82 ± 0.91*	4.23 ± 1.38^#^	3.91 ± 0.91^△^	4.48 ± 1.51^▲^
Activity disturbances	6.51 ± 0.28	6.48 ± 0.71	6.53 ± 0.41	6.74 ± 0.85	6.61 ± 1.37*	7.34 ± 1.93^#^	6.69 ± 1.58^△^	7.76 ± 1.78^▲^
Aggressiveness	5.06 ± 0.30	5.02 ± 0.39	5.13 ± 1.02	5.41 ± 0.99	5.36 ± 1.32*	6.02 ± 1.59^#^	5.52 ± 1.46^△^	6.48 ± 1.91^▲^
Diurnal rhythm disturbances	2.61 ± 0.33	2.59 ± 0.47	2.76 ± 0.83	3.15 ± 0.42	3.11 ± 0.76	3.55 ± 0.92^#^	3.24 ± 0.89	3.84 ± 0.78^▲^
Affective disturbances	3.15 ± 0.23	3.18 ± 0.35	3.23 ± 0.59	3.31 ± 0.35	3.34 ± 0.64	3.88 ± 0.72^#^	3.41 ± 0.58	4.17 ± 0.86^▲^
Anxieties and phobias	2.22 ± 0.33	2.19 ± 0.52	2.38 ± 0.41	2.69 ± 0.58	2.57 ± 0.57*	3.02 ± 0.38^#^	2.71 ± 0.46^△^	3.36 ± 0.47^▲^
NPI mean score								
Delusions	1.54 ± 0.59	1.53 ± 0.54	1.58 ± 0.61	1.82 ± 0.57	1.75 ± 0.89*	2.13 ± 0.96^#^	1.86 ± 1.02^△^	2.41 ± 1.62^▲^
Hallucinations	3.72 ± 0.59	3.79 ± 0.61	3.75 ± 1.48	3.95 ± 0.61	3.82 ± 0.91*	4.23 ± 1.38^#^	3.91 ± 0.85^△^	4.59 ± 1.61^▲^
Agitation	5.32 ± 0.91	5.41 ± 0.46	5.45 ± 0.96	6.48 ± 0.88	6.28 ± 1.09*	7.48 ± 0.88^#^	6.89 ± 1.36^△^	7.97 ± 1.53^▲^
Depression	4.21 ± 0.81	4.23 ± 0.16	4.26 ± 1.01	4.19 ± 0.72	4.19 ± 1.42	4.29 ± 0.38	4.37 ± 1.32	4.46 ± 0.81
Anxiety	2.34 ± 0.69	2.33 ± 0.41	2.38 ± 0.97	2.42 ± 0.86	2.43 ± 1.13	2.46 ± 1.04	2.39 ± 1.65	2.51 ± 1.42
Euphoria	3.63 ± 0.62	3.62 ± 0.68	3.73 ± 0.89	3.72 ± 0.56	3.75 ± 0.61	3.80 ± 0.69	3.81 ± 0.35	3.83 ± 0.92
Apathy	3.31 ± 0.72	3.29 ± 0.48	3.29 ± 0.84	3.33 ± 0.83	3.39 ± 0.97	3.27 ± 0.66	3.43 ± 0.77	3.39 ± 0.69
Disinhibition	2.74 ± 0.57	2.79 ± 0.49	2.76 ± 0.36	2.91 ± 0.55	2.73 ± 0.69	2.89 ± 0.58	2.81 ± 0.37	2.85 ± 0.84
Ignitability	3.26 ± 0.75	3.27 ± 0.77	3.29 ± 0.82	4.17 ± 0.52	3.85 ± 0.76	4.08 ± 0.83	3.72 ± 0.53	3.98 ± 0.56
Aberrant motor behavior	4.28 ± 0.69	4.25 ± 0.87	4.27 ± 0.74	4.63 ± 0.73	4.26 ± 1.09*	5.12 ± 1.26^#^	4.31 ± 0.96^△^	5.33 ± 1.09^▲^
Sleep disturbance	5.32 ± 0.83	5.25 ± 0.68	5.38 ± 1.23	6.07 ± 0.79	5.87 ± 1.73*	6.69 ± 1.28^#^	5.91 ± 1.65^△^	7.06 ± 1.37^▲^
Appetite	4.08 ± 0.57	4.11 ± 0.36	4.12 ± 0.87	4.28 ± 0.31	4.18 ± 0.39	4.17 ± 0.61	4.24 ± 0.57	4.03 ± 0.55
DFA of actigraph activity								
Diurnal activity	0.84 ± 0.11	0.83 ± 0.24	0.83 ± 0.19	0.84 ± 0.17	0.85 ± 0.15	0.88 ± 0.13	0.84 ± 0.19	0.89 ± 0.21
Evening activity	0.85 ± 0.12	0.86 ± 0.09	0.86 ± 0.13	0.91 ± 0.14	0.86 ± 0.35*	0.95 ± 0.11^#^	0.88 ± 0.27^△^	0.98 ± 0.23^▲^
Nocturnal activity	0.92 ± 0.13	0.91 ± 0.14	0.94 ± 0.15	0.99 ± 0.16	0.95 ± 0.16*	1.08 ± 0.15^#^	0.97 ± 0.13^△^	1.28 ± 0.17^▲^

*P < 0.05, compared with week 20 of placebo group; ^#^ *P* < 0.05, compared with week 0 of placebo group; ^△^ *P* < 0.05, compared with week 30 of placebo group; ^▲^ *P* < 0.05, compared with week 0 of placebo group.
